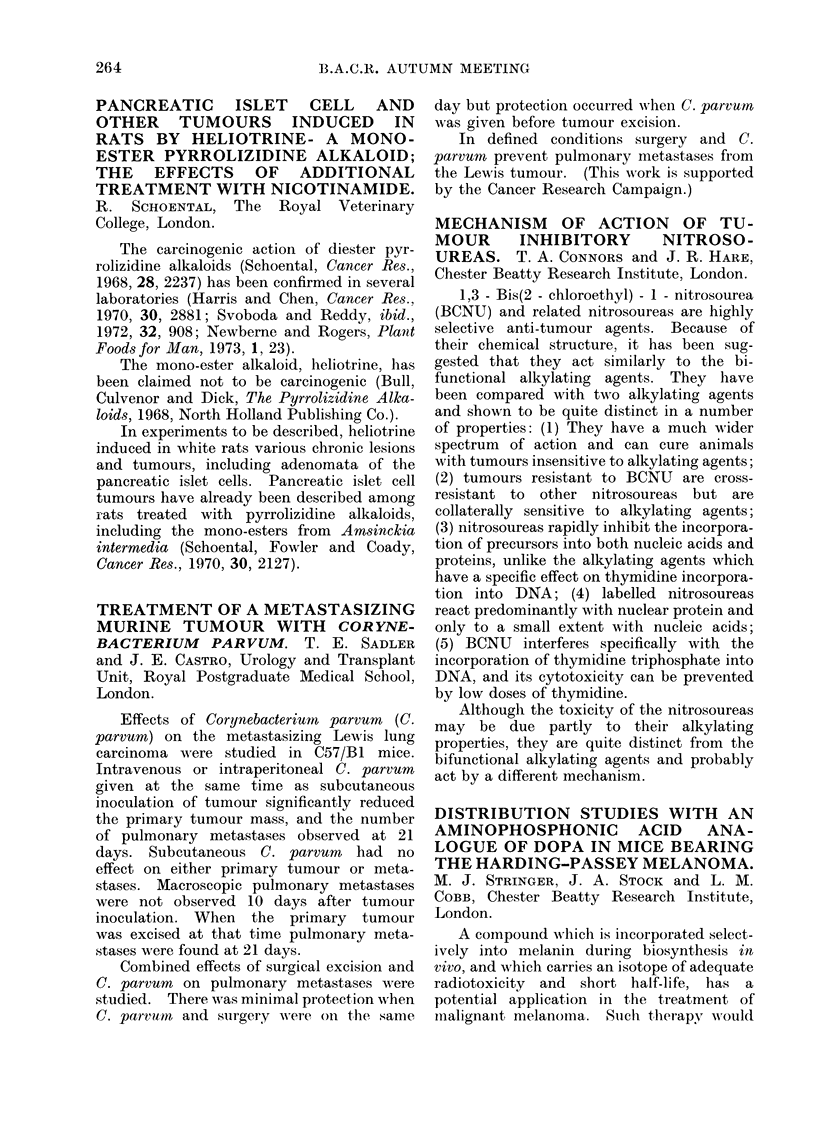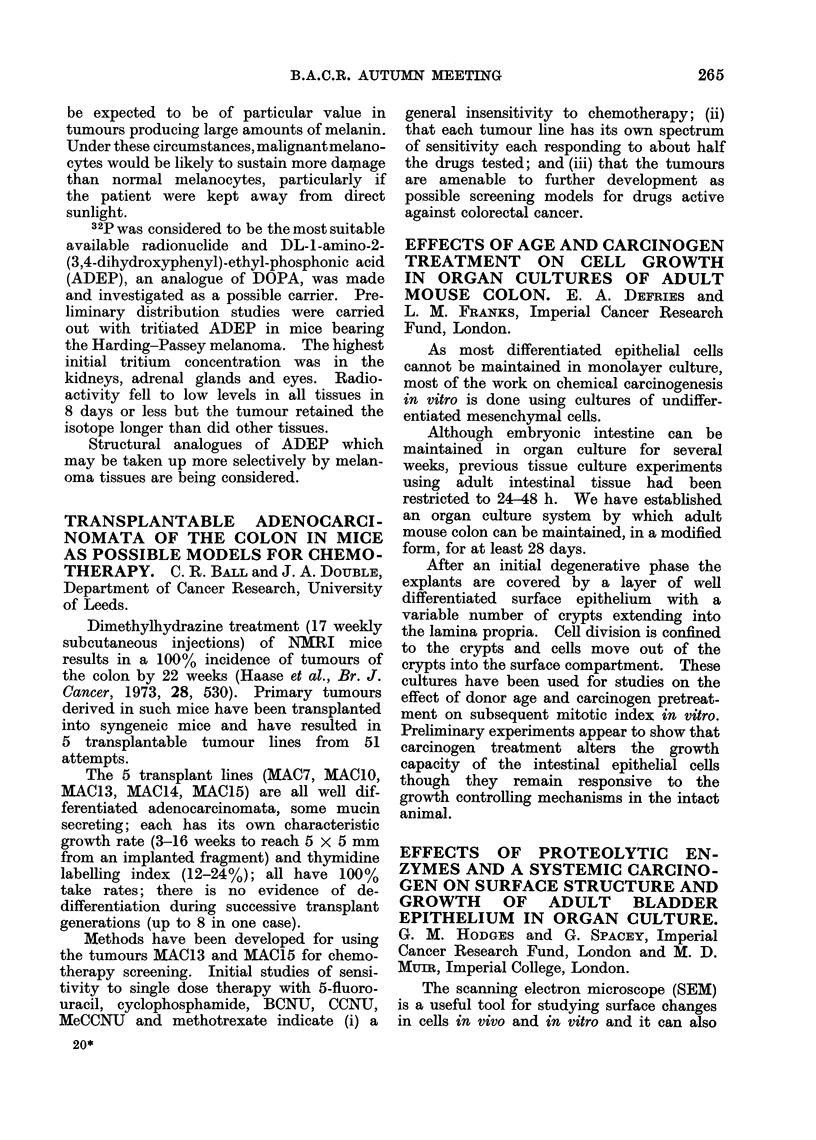# Proceedings: Distribution studies with an aminophosphonic and analogue of DOPA in mice bearing the Harding-Passey melanoma.

**DOI:** 10.1038/bjc.1975.52

**Published:** 1975-02

**Authors:** M. J. Stringer, J. A. Stock, L. M. Cobb


### DISTRIBUTION STUDIES WITH AN AMINOPHOSPHONIC ACID ANALOGUE OF DOPA IN MICE BEARING THE HARDING–PASSEY MELANOMA.

M. J. STRINGER, J. A. STOCK and L. M. COBB, Chester Beatty Research Institute, London.

A compound which is incorporated selectively into melanin during biosynthesis *in vivo*, and which carries an isotope of adequate radiotoxicity and short half-life, has a potential application in the treatment of malignant melanoma. Such therapy would be expected to be of particular value in tumours producing large amounts of melanin. Under these circumstances, malignant melanocytes would be likely to sustain more damage than normal melanocytes, particularly if the patient were kept away from direct sunlight.

$^{32}$P was considered to be the most suitable available radionuclide and DL-1-amino-2-(3,4-dihydroxyphenyl)-ethyl-phosphonic acid (ADEP), an analogue of DOPA, was made and investigated as a possible carrier. Preliminary distribution studies were carried out with tritiated ADEP in mice bearing the Harding–Passey melanoma. The highest initial tritium concentration was in the kidneys, adrenal glands and eyes. Radioactivity fell to low levels in all tissues in 8 days or less but the tumour retained the isotope longer than did other tissues.

Structural analogues of ADEP which may be taken up more selectively by melanoma tissues are being considered.